# Meningococcal Seroepidemiology 1 Year After the PsA-TT Mass Immunization Campaign in Burkina Faso

**DOI:** 10.1093/cid/civ519

**Published:** 2015-11-09

**Authors:** Haoua Tall, Seydou Yaro, Hervé B. N. Kpoda, Soumeya Ouangraoua, Caroline L. Trotter, Berthe-Marie Njanpop Lafourcade, Helen Findlow, Xilian Bai, Catherine Martin, Ikenna Nwakamma, Jean Bosco Ouedraogo, Bradford D. Gessner, Ray Borrow, Judith E. Mueller

**Affiliations:** 1 Agence de Médecine Préventive, Paris, France; 2 Centre Muraz, Bobo-Dioulasso, Burkina Faso; 3 Disease Dynamics Unit, Department of Veterinary Medicine, University of Cambridge; 4 Vaccine Evaluation Unit, Public Health England, Manchester Royal Infirmary, United Kingdom; 5 Ecole des Hautes Etudes en Santé PubliqueFrench School of PublicHealth, Sorbonne Paris Cité, France; 6 Institut de Recherche en Science et Santé, Bobo-Dioulasso, Burkina Faso; 7 Institut Pasteur, Paris, France

**Keywords:** sub-Saharan Africa, conjugate, *Neisseria meningitidis*, group A, seroepidemiologic studies

## Abstract

***Background.*** A group A meningococcal (MenA) conjugate vaccine, PsA-TT (MenAfriVac), was introduced in Burkina Faso via mass campaigns between September and December 2010, targeting the 1- to 29-year-old population. This study describes specific antibody titers in the general population 11 months later and compares them to preintroduction data obtained during 2008 using the same protocol.

***Methods.*** During October–November 2011, we recruited a representative sample of the population of urban Bobo-Dioulasso aged 6 months to 29 years, who underwent standardized interviews and blood draws. We assessed anti-MenA immunoglobulin G (IgG) concentrations (n = 200) and, using rabbit complement, serum bactericidal antibody (SBA) titers against 2 group A strains: reference strain F8238 (SBA_ref_) (n = 562) and strain 3125 (SBA_3125_) (n = 200).

***Results.*** Among the 562 participants, 481 (86%) were aged ≥23 months and had been eligible for the PsA-TT campaign. Among them, vaccine coverage was 86.3% (95% confidence interval [CI], 82.7%–89.9%). Prevalence of putatively protective antibodies among vaccine-eligible age groups was 97.3% (95% CI, 95.9%–98.7%) for SBA_ref_ titers ≥128, 83.6% (95% CI, 77.6%–89.7%) for SBA_3125_ ≥128, and 84.2% (95% CI, 78.7%–89.7%) for anti-MenA IgG ≥2 µg/mL. Compared to the population aged 23 months to 29 years during 2008, geometric mean titers of SBA_ref_ were 7.59-fold higher during 2011, 51.88-fold for SBA_3125_, and 10.56-fold for IgG.

***Conclusions.*** This study shows high seroprevalence against group A meningococci in Burkina Faso following MenAfriVac introduction. Follow-up surveys will provide evidence on the persistence of population-level immunity and the optimal vaccination strategy for long-term control of MenA meningitis in the African meningitis belt.

To eliminate meningococcal meningitis epidemics in the African meningitis belt, a group A meningococcal (MenA) conjugate vaccine, PsA-TT (MenAfriVac), was developed by the Meningitis Vaccine Project and subsequently introduced in this region starting in Burkina Faso during 2010. Vaccine was administered during mass campaigns targeting the 1- to 29-year-old population. The vaccine has shown good immunogenicity in clinical trials [1], and current surveillance data suggest a strong reduction of MenA disease burden [2]; however, the duration of persistence of vaccine-induced antibody and, therefore, the duration of protection against disease and carriage are not known. Age-specific estimates of antibody persistence are required to develop appropriate long-term vaccination strategies, including introduction in the Expanded Programme on Immunization, or repeat mass campaigns in specific age groups.

We therefore aim to evaluate anti-MenA antibody persistence after PsA-TT mass campaigns in Burkina Faso, by conducting 3 seroprevalence surveys in the general population over a period of 5 years. We previously have collected data on population-level anti-meningococcal immunity in Bobo-Dioulasso, Burkina Faso, during 2008 [3], which will serve for baseline comparisons. The objectives of this article are to document population-level anti-MenA seroprevalence in Bobo-Dioulasso during 2011, 1 year after the PsA-TT mass campaign, to compare with population-level immunity before PsA-TT introduction, and to describe individual risk factors for low antibody titers following PsA-TT vaccination.

## METHODS

During October–November 2011, 10–11 months after the PsA-TT mass campaign, we conducted a cross-sectional study on a representative sample of the urban population of Bobo-Dioulasso aged 6 months to 29.9 years. The study protocol was approved by the National Ethics and Research Committee of Burkina Faso, and the Ethics Committee of Centre Muraz, Bobo-Dioulasso, Burkina Faso. Participants or their legal guardian (if aged <18 years) signed an informed consent document before enrollment or provided a fingerprint in presence of an independent witness (if illiterate). To be eligible for inclusion, individuals had to be a resident of urban Bobo-Dioulasso and not report any serious illness, including severe malnutrition or bleeding disorder. We recruited participants in 8 age groups: 6–11 months and 12–22 months (not born or not eligible for PsA-TT in December 2010; each contributing 7.5% to the total number of participants); 1.9 to <3 years, 3 to <5 years, 5 to <10 years, 10 to <15 years, 15 to <20 years, 20 to <30 years (all eligible for PsA-TT during December 2010; each contributing 14% to the total number of participants). Sample sizes were planned to allow describing a statistically significant decline by 20% in each vaccine-eligible age group from one survey to the other (this article reports the first survey).

Participants were identified as follows. The 25 residential sectors of the urban area of Bobo-Dioulasso were regrouped into 10 geographic areas. Within each area, sampling starting points were randomly selected from an exhaustive list of numbered crossroads, weighing for area population size, based on health center catchment population estimates. For each starting point, a compound was randomly identified. In each compound, at most 1 person could be recruited from age ranges <5 years, 5–14 years, and 15–29 years. Among the residents of the compound corresponding to the required age range, the participants were randomly drawn. The identified person, or his or her legal guardian for children <18 years, gave written consent to participate in the study. If the selected person refused or if no person in the desired age group resided in the compound, the compound opposite to the left was visited and the procedure repeated. Participants were invited to the Centre Muraz outpatient clinic for the study visit a few days later. During the study visit, study nurses took height and weight for children aged <9 years and administered a standardized questionnaire on sociodemographic information, relevant medical history, and living conditions. Meningococcal vaccination status was assessed based on vaccination card or health booklet, recall specifically for MenAfriVac, or any “meningitis vaccine” given during a mass campaign during 2010 (no other campaigns were conducted in Bobo-Dioulasso during 2010). A 2- to 5-mL blood sample was taken from each participant.

Blood samples were stored at 8°C until centrifugation up to 4 hours later. Following centrifugation, sera were frozen at −80°C until analysis. Serum bactericidal antibody (SBA) titers were measured using rabbit complement against 2 MenA strains: the reference strain F8238 of immunotype L11 (SBA_ref_, all sera) and an alternative MenA strain 3125 of immunotype L10 (SBA_3125_, 200 randomly selected serum samples) [4]. MenA–specific immunoglobulin G (IgG) concentration was determined by enzyme-linked immunosorbent assay [5]. All serological analyses were performed at the Vaccine Evaluation Unit, Manchester, United Kingdom.

We defined malnutrition as weight for length or height <3rd percentile among <6-year-old children, and as weight for age <3rd percentile among 6- to 8-year-old children, using World Health Organization growth standards [6].

We calculated geometric means of SBA titers and IgG concentrations with 95% confidence intervals (CIs). Based on previous seroepidemiologic results in this population [3, 7], we used SBA titers ≥128 or ≥1024 as cutoffs for seroprevalence estimation in the population. We evaluated the correlation between SBA_ref_ and SBA_3125_ titers using Spearman rank correlation testing the coefficient ρ.

Using logistic regression, we estimated the age-specific association (odds ratio [OR]) between PsA-TT vaccination status (recall or document-based) and high SBA titers (SBA_ref_ ≥ 128 and ≥1028) among all participants. We also evaluated whether individual and household characteristics were risk factors for SBA_ref_ <128 or SBA_ref_ <1028 among persons vaccinated with MenAfriVac 1 year earlier. For these analyses, variables showing an association with the outcome variable at *P* < .2 in univariate or age-adjusted analyses were included in a full logistic regression model, which then was reduced in a stepwise backward manner to a parsimonious model including only variables contributing at *P* < .05.

Results were compared to data from a seroprevalence survey conducted in the same population using a similar protocol during January–February 2008, 2.75 years before the PsA-TT mass campaign [3]. This study had used a comparable protocol and sampling scheme, and serological analyses were performed in the same laboratory.

All analyses were performed using Stata software version 12/IC and adjusting for design effect using the *svy* commands (except for Spearman correlation).

## RESULTS

### Vaccination Status

Among the 562 participants, 481 (86%) were aged between 23 months and 29 years and had been eligible for the PsA-TT campaign. All of them provided information on PsA-TT vaccination status; 455 (95%) had resided in Bobo-Dioulasso and 472 (98%) in Burkina Faso during 2010. Two persons aged 4 and 17 years reported intermittent residency outside Burkina Faso and no PsA-TT vaccination.

Of PsA-TT eligible participants, 415 recalled receiving MenAfriVac or a “meningitis vaccine” received during a campaign in 2010, independent of document confirmation. PsA-TT coverage was ≥86.3% (95% confidence interval [CI], 82.7%–89.9%) among those vaccine-eligible individuals who were resident in Bobo-Dioulasso during 2010 (88.8% [95% CI, 85.6%–92.0%]) compared with those who lived elsewhere that year (n = 24; 44.0% [95% CI, 16.9%–71.1%]). PsA-TT vaccination was document-confirmed for 255 eligible residents (56%) and 4 nonresidents (16%). None of the 7 persons indicating 2010 residence outside Burkina Faso reported vaccination.

PsA-TT vaccination was reported or confirmed by vaccination card for 5 and 2, respectively, of the 42 children aged 11–22 months (11.9% and 7.1%, respectively), who had been born but were too young to be eligible during the 2010 campaign.

**Figure 1.**
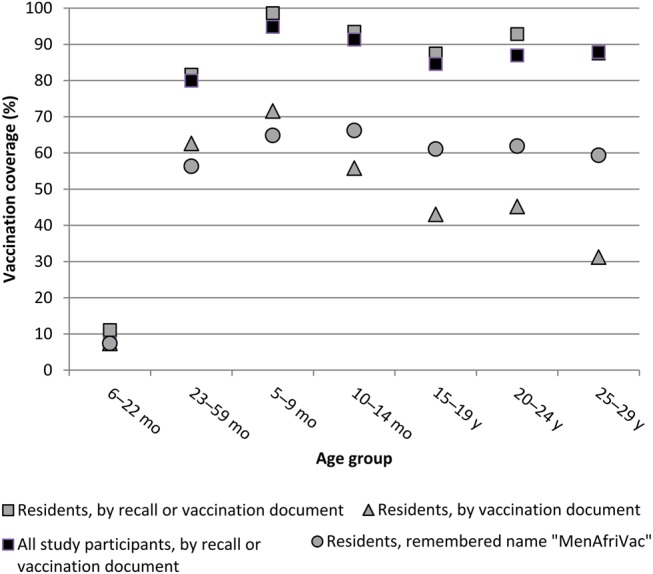
Age-specific vaccination coverage with meningococcal group A conjugate vaccine in Bobo-Dioulasso, Burkina Faso, 2011. Estimates are given for all study participants, and specifically for participants who were residents of Bobo-Dioulasso in 2010 according to different information types.

Vaccination coverage did not vary substantially by age group (Figure 1). Recall- or document-based vaccination among all participants was lowest among children aged 23–59 months (80.0%) and highest among children aged 5–9 years (94.9%). Approximately 60% of participants remembered specifically receiving the MenAfriVac vaccine, with little variation by age, whereas document-confirmed vaccination declined from 71.6% among 5- to 9-year-old children to 31.3% among ≥25-year-old adults.

### Seroprevalence

**Figure 2.**
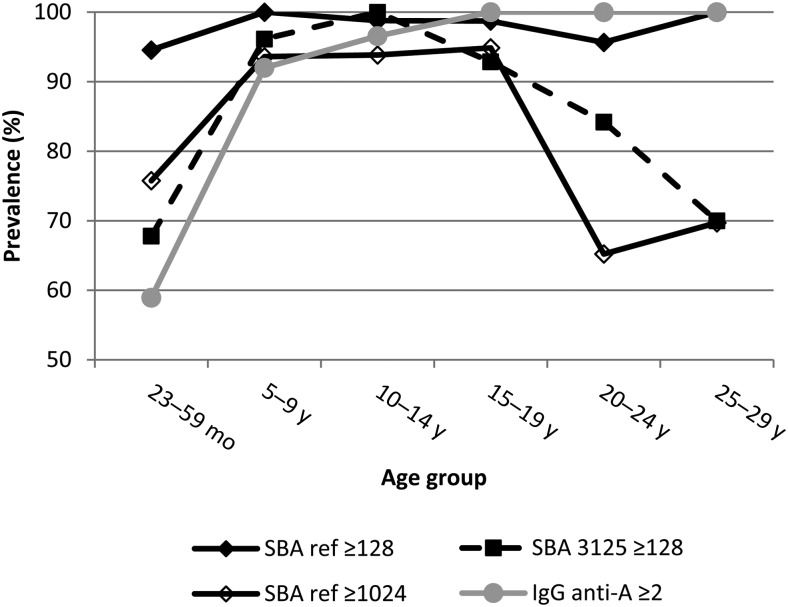
Seroprevalence according to different serologic outcomes among the population aged 23 months to 29 years, by age group, Bobo-Dioulasso, Burkina Faso, 2011. Included are all 481 study participants who were eligible for meningococcal group A conjugate vaccination during 2010. Abbreviations: anti-A, against meningococcal serogroup A; IgG, immunoglobulin G; SBA, serum bactericidal antibody.

Among the 481 participants aged 23 months to 29 years, the SBA_ref_ geometric mean titer (GMT) was 1939 (95% CI, 1700–2212), the SBA_3125_ GMT was 375 (95% CI, 261–538), and the anti-MenA IgG geometric mean concentration was 28.12 µg/mL (95% CI, 21.76–32.80 µg/mL). Prevalence of SBA_ref_ ≥ 128 was 97.3% (95% CI, 95.9%–98.7%); of SBA_ref_ ≥ 1024, 83.4% (95% CI, 80.0%–86.8%); of SBA_3125_ ≥ 128, 83.6% (95% CI, 77.6%–89.7%); and of IgG ≥ 2 µg/mL, 84.2% (95% CI, 78.7%–89.7%). Seroprevalence of SBA_ref_ ≥ 128 did not vary by age, whereas prevalence of SBA_ref_ ≥ 1024 and SBA_3125_ ≥ 128 was close to 100% among 5- to 19-year-olds and as low as 65% among 23- to 59-month-olds and 20- to 29-year-olds (Figure 2). Prevalence of IgG ≥ 2 µg/mL increased by age, from 59% among <5-year-olds to 100% among adults.

All GMT and seroprevalence estimates in participants with documented PsA-TT vaccination were higher than in the entire population (Table 1). The association (OR) between vaccination and SBA_ref_ ≥ 1024 was 2.20 (95% CI, .29–16.84) among 11- to 22-month-old children, 1.48 (95% CI, .36–3.49) for those aged 23–59 months, 3.89 (95% CI, .70–21.72) for children aged 5–14 years, and 1.73 (95% CI, .57–5.29) for participants aged 15–29 years. Too few samples were tested for SBA_3125_ and IgG for statistical analyses.

**Table 1.** Immune Status in the General Population of Bobo-Dioulasso, Burkina Faso, 2011, by Age Group

Parameter	2011						2008		
	All Participants			Participants With Confirmed MenAfriVac Vaccination			All Participants		
	23–59 mo	5–14 y	15–29 y	23–59 mo	5–14 y	15–29 y	23–59 mo	5–14 y	15–29 y
SBA reference strain	n = 165	n = 159	n = 157	n = 101	n = 98	n = 60	n = 110	n = 228	n = 341
GMT (95% CI)	1397 (1052–1856)	3352 (2825–3976)	1571 (1287–1919)	1600 (1103–2320)	3606 (2937–4429)	1552 (1015–2374)	160 (93–274)	231 (164–325)	338 (266–430)
% ≥128 (95% CI)	94.5 (91.1–98.0)	99.4 (98.1–100)	98.1 (95.9–100)	95.0 (90.7–99.4)	100^a^	96.7 (92.0–100)	70.9 (62.3–79.5)	78.1 (72.7–83.6)	83.3 (79.4–87.2)
% ≥1024 (95% CI)	75.8 (69.6–81.9)	93.7 (90.0–97.4)	80.9 (74.9–86.9)	78.2(70.2–86.2)	93.9 (89.1–98.7)	80.0 (68.5–91.5)	38.2 (29.0–47.4)	43.9 (37.4–50.4)	44.3 (38.80–49.7)
SBA strain 3125	n = 59	n = 55	n = 57	n = 36	n = 31	n = 24	n = 110	n = 228	n = 341
GMT (95% CI)	124 (60–253)	1237 (868–1763)	373 (209–668)	158 (70–358)	1498 (850–2638)	542 (238–1235)	3 (2–4)	5 (4–6)	13 (10–17)
% ≥128 (95% CI)	67.8 (55.7–79.8)	98.2 (94.5–100)	86.0 (76.4–95.6)	72.2 (57.9–86.5)	96.8 (90.2–100)	91.7 (79.4–100)	7.3 (2.3–12.2)	14.0 (9.5–18.6)	30.1 (24.4–34.9)
IgG anti-MenA	n = 58	n = 56	n = 57	n = 36	n = 32	n = 24	n = 110	n = 228	n = 341
GMC (95% CI)	5.6 (3.5–7.7)	28.0 (16.9–39.2)	51.1 (37.9–64.3)	6.4 (3.4–9.4)	33.2 (16.2–50.2)	57.2 (36.2–78.1)	1.75 (1.47–2.03)	7.24 (4.80–9.67)	21.71 (16.74–26.67)
% ≥2 µg/mL (95% CI)	60.3 (48.3–72.4)	92.9 (85.8–99.9)	100^b^	72.2 (57.3–87.2)	93.8 (84.8–100)	100^c^	29.5 (20.9–38.0)	58.3 (52.2–64.3)	93.3 (90.7–96.0)

The table compares SBA titers, IgG anti-A concentrations, and seroprevalence during 2011 (10–11 months after PsA-TT mass campaign) among all participants and specifically those for whom PsA-TT vaccination was confirmed by a vaccination document, and to 2008 preintroduction data collected in the same study population [3].

Abbreviations: CI, confidence interval; GMC, geometric mean concentration; GMT, geometric mean titer; IgG, immunoglobulin G; MenA, group A meningococcal; SBA, serum bactericidal antibody.

95% CI not estimated with correction for design effect; without correction for design effect: ^a^ 96.3%–100%, ^b^ 85.8%–100%, ^c^ 93.7%–100%.

Compared with the population aged 23 months to 29 years of Bobo-Dioulasso during 2008, geometric mean titers of SBA_ref_ were 7.59-fold higher during 2011, 51.88-fold higher for SBA_3125_, and 10.56-fold higher for IgG. Seroprevalence of SBA_ref_ ≥ 128 and SBA_ref_ ≥ 1024 was 1.23-fold and 1.94-fold higher, respectively, during 2011, 3.92-fold higher for SBA_3125_, and 1.18-fold higher for IgG.

For all serologic outcomes (geometric means or seroprevalence of SBA_ref_, SBA_3125_, and IgG) among participants aged 23 months to 29 years, values were higher among 2011 participants with documented PsA-TT vaccination compared with 2008 participants (Table 1).

Among vaccinated persons (document-based), the correlation between SBA_ref_ and SBA_3125_ titers was strong among children aged 23 months to 4 years (ρ = 0.81, *P* < .001), and moderate for 5- to 14-year-olds (ρ = 0.66, *P* < .001) and 15- to 29-year-olds (ρ = 0.62, *P* = .001). IgG concentrations correlated poorly with SBA_ref_ titers, whereas they correlated fairly well with SBA_3125_ titers among <5-year-old (ρ = 0.56, *P* < .001) and 5- to 14-year-old children (ρ = 0.60, *P* < .001). Among persons aged ≥15 years, IgG did not correlate with any SBA. In the 2008 survey, correlations between levels of SBA_ref_, SBA_3125_, and IgG had been poor (ρ < 0.53) across outcome combinations and age groups (Table 2).

**Table 2.** Correlation Between Serological Outcomes, by Age Group, Comparing a Population Certainly Vaccinated With PsA-TT 11 Months Earlier Versus a Certainly Unvaccinated Population of the Same Age, Bobo-Dioulasso, Burkina Faso, 2008 and 2011

Age Group	2011 (PsA-TT, MenAfriVac Confirmed by Document, n = 89)			2008 (All Participants, n = 711)		
	SBA_ref_–SBA_3125_	SBA_ref_–IgG	SBA_3125_–IgG	SBA_ref_–SBA_3125_	SBA_ref_–IgG	SBA_3125_–IgG
23 mo–4 y	0.82 (<.001)	0.33 (.055)	0.54 (.001)	0.22 (.008)	0.03 (.759)	0.14 (.093)
5–14 y	0.66 (<.001)	0.40 (.026)	0.59 (<.001)	0.30 (<.001)	−0.004 (.951)	0.23 (.001)
15–29 y	0.62 (.001)	0.07 (.746)	0.02 (.929)	0.33 (<.001)	0.09 (.098)	0.53 (<.001)

Data are shown as Spearman ρ (*P* value).

Abbreviations: IgG, immunoglobulin G; SBA, serum bactericidal antibody.

Source: [3].

### Determinants of Low SBA Titers

Among the 421 participants with document-based or recalled PsA-TT vaccination (of any age), 199 (47%) were men, 78 (19%) reported anti-meningococcal vaccination before 2010, 336 (80%) had a television set in the compound, 163 (39%) spent 1 hour or more daily exposed to kitchen fire smoke, 199 (47%) lived in a compound with >4 persons per room, and 14 (7%) of children <9 years of age were malnourished.

In univariate analyses, age, sex, smoke exposure, and malnutrition were associated with SBA_ref_ < 128 or SBA_ref_ < 1024 and were therefore included in full multivariate models. In the resulting parsimonious multivariate models, SBA_ref_ < 128 was associated with age <5 years (OR, 9.99 [95% CI, 1.90–52.5]) (reference age, 15–29 years; the age group 5–14 years could not be evaluated due to small sample size) and with daily exposure to kitchen fire smoke of ≥1 hour (OR, 5.65 [95% CI, 1.42–22.46]). Similarly, SBA_ref_ < 1024 was associated with kitchen smoke exposure ≥1 hour (OR, 2.22 [95% CI, 1.21–4.05]) and age <5 years (OR, 2.20 [95% CI, 1.16–4.17]), whereas the age group 5–14 years was less likely to have low titers (OR, 0.35 [95% CI, .14–0.85]). Apart from age, no risk factors could be identified for low SBA_3125_ titers.

Among vaccinated children aged <9 years, only kitchen smoke exposure was a risk factor for SBA_ref_ < 1024 in the final multivariate model (OR, 2.61 [95% CI, 1.30–5.23]). The OR for malnutrition was 3.08 (95% CI, .81–11.72) if forced into the model. SBA_ref_ < 128 could not be evaluated due to small sample size.

## DISCUSSION

This study is the first serological evaluation of the impact of PsA-TT mass campaigns in the African meningitis belt. In an urban population of Burkina Faso, we found high SBA_ref_ GMTs of almost 2000, with >90% prevalence of SBA_ref_ > 128. Compared to a preintroduction evaluation in the same population in 2008, SBA_ref_ GMTs had increased 8-fold, although the prevalence of SBA titers >1024 had increased only <2-fold. However, an alternative SBA assay using strain 3125 showed 50-fold higher GMTs and 4-fold higher seroprevalence of titers >128 compared with the preintroduction survey.

In the 2008 study, conducted 2 years before PsA-TT introduction, SBA_3125_ GMTs were about 20-fold lower for SBA_3125_ than for SBA_ref_. The interpretation was that SBA_3125_ reflects more specifically immunity directed against capsular polysaccharides (and thus immunity induced by polysaccharide-based vaccines), whereas SBA_ref_ reflects overall immunity against the bacterium, and thus natural immunity [3]. The observations that SBA_ref_ and SBA_3125_ correlated much more strongly in the 2011 survey after PsA-TT vaccination compared with the 2008 preintroduction survey, and that the difference in seroprevalence between the 2008 and 2011 surveys was much more pronounced for SBA_3125_ than for SBA_ref_, confirm this interpretation. Poolman et al have suggested that SBA against strain 3125 (lipooligosaccharide immunotype L10) is a more specific measure of vaccine-induced immunity because it does not capture bactericidal action of antibodies directed against L11 (present in the F3828 reference strain), as the latter could result from natural exposure during carriage, while it is equally sensitive to antibody induced by vaccination with MenA polysaccharide–containing vaccines [8]. The underlying assumption is that immune response to natural lipooligosaccharide immunotype L11 is stronger than that to natural MenA capsular polysaccharide. We suggest that SBA_3125_ should be included in studies evaluating the immune response to PsA-TT. For example, a recent study of PsA-TT trial data in The Gambia evaluated the impact of preexisting antibody on immune responses to PsA-TT, relying solely on SBA_ref_ [9]. The authors described that 4-fold increase of SBA_ref_ was less frequent among those vaccines with higher preexisting SBA_ref_, suggesting that natural immunity could impact negatively immune response to vaccination, which would have consequences for vaccination strategies. Our findings emphasize the need to evaluate SBA_3125_ in such studies to enable full interpretation.

At an individual level, 2011 PsA-TT vaccination status was only moderately associated with immune status, including SBA_3125_, except for youngest children (vaccinated despite being outside the target group). Given results from clinical trials [1] showing excellent immunogenicity, it may be that PsA-TT coverage (86.3%) has been underestimated in our study. In a neighboring region in Burkina Faso, a survey showed PsA-TT coverage ranging from 71% (overall, card) and 89% (urban population, recall) to 94% (overall, recall) [10]. Our estimates in an urban population based on recall or documentation are similar, whereas confirmation by vaccination card is poorer. In consequence, some of the persons classed as unvaccinated with high antibody titers may have received PsA-TT. This explanation is supported by the fact that preintroduction seroprevalence during 2008 was substantially lower than that among the unvaccinated population in the present study (data not shown). This, again, could in theory be due to a more intensive circulation of MenA in the population during 2009–2011 vs the years before 2008, which is, however, not supported by the epidemiological data. During 2006–2008, an epidemic wave occurred in the Bobo-Dioulasso region, whereas since 2009, few cases have been observed (Ministry of Health, Burkina Faso). The comparison between “vaccinated” and “unvaccinated” persons in our study should therefore be interpreted with caution.

A surprising association with lower antibody levels was found for daily prolonged exposure to kitchen fire smoke. This exposure has been described as a risk factor for meningococcal meningitis during epidemics [7, 11] and for pneumonia [12]. Although facilitated mucosal invasion is in general held as the mechanism for this increased disease risk, the association with immune status (through weaker immune response or more quickly waning antibody) may also contribute. As this exposure was frequent in the population, further research should identify the pathophysiological mechanism, evaluate the maximal tolerable duration of exposure, and develop socially acceptable recommendations and technologies to reduce exposure. Beyond group- and species-specific vaccine prevention, some disease reduction may be obtained through such a pathogen-unspecific approach.

Since the mass campaign in 2010 until today, no MenA case has been confirmed in Bobo-Dioulasso (Weekly reports 2011–2015 by the Ministry of Health, Burkina Faso [“Situation épidémiologique hebdomadaire,” Service de surveillance épidémiologique, Direction de la lutte contre la maladie]). This 4-year period is longer than the previously observed period of MenA absence during 2002–2003 [13], which suggests that the population-level anti-MenA seroprevalence we report here provides protection against MenA. The vaccine may be working in several different ways, and we do not yet fully understand the relative contributions of direct individual protection from serum antibodies, individual protection from mucosal immunity preventing any nasopharyngeal infection, or the population-level indirect effects resulting from much reduced circulation of the MenA. The latter is suggested by large carriage studies in Burkina Faso and Chad showing reduced MenA carriage following vaccine introduction [2, 14]. Mucosal immunity is probably conditional on very high titers of serum antibody, which exudate onto the nasopharyngeal mucosa. The high serum titers observed in our study, within 1 year of vaccination, may well be sufficient for mucosal immunity, as well as direct protection against invasion and disease, although correlates of protection have not been definitively established. Eventually, waning antibodies could allow pathogen transmission to become reestablished in the population, with the associated risk of outbreaks in new birth cohorts, age groups not targeted by the 2010 campaign, or migrant populations. The definition of correlates of protection against disease and carriage (although particularly challenging for mucosal immunity) would be an important step in our understanding of the mechanism of vaccine protection. Further evidence from repeated large carriage studies and exhaustive meningitis surveillance, in combination with our seroprevalence estimates, will also provide indirect evidence on this question.

Data on another MenA conjugate vaccine trialed in Denmark suggest that group-specific SBA titers in young adults decrease by 60% during the first 3 years after vaccination [15]. The titers we observed 1 year after vaccination likely already include a part of this initial waning. Further data from 2013 and 2015 will provide evidence on the rate at which this waning continues over the longer term. Phase 2 immunogenicity trials in Mali and The Gambia [1] will also conduct follow-up of children up to 3 years postvaccination to evaluate antibody persistence. These studies reflect vaccination under trial conditions. Our population-level estimates include factors such as vaccine coverage, population migration, and reduced natural boosting (following MenA elimination), which will influence the effective impact of PsA-TT mass campaigns and which need to be assessed outside clinical trials.

In conclusion, this study shows high anti-meningococcal seroprevalence in Burkina Faso following PsA-TT introduction. Upcoming surveys will measure the persistence of this population-level immunity and inform the optimal vaccination strategies for long-term control of group A meningococcal meningitis in the African meningitis belt.
